# Spotting childhood abdominal tumours: a systematic review and meta-analysis of the clinical presentation

**DOI:** 10.1136/archdischild-2025-329097

**Published:** 2025-10-05

**Authors:** Lorna Ni Cheallaigh, Jo-Fen Liu, Ashley Ball-Gamble, David Walker, Timothy A Ritzmann, Dhurgshaarna Shanmugavadivel

**Affiliations:** 1 UCL GOS Institute of Child Health, London, UK; 2 CCLG: The Children & Young People’s Cancer Association, Leicester, UK; 3 Children’s Brain Tumour Research Centre, University of Nottingham, Nottingham, UK; 4 Lifespan and Population Health, University of Nottingham School of Medicine, Nottingham, England, UK

**Keywords:** Child Health, Paediatrics

## Abstract

**Background:**

We performed a systematic review and meta-analysis to identify pre-diagnostic symptoms/signs for childhood abdominal tumours to inform ongoing efforts to achieve earlier diagnoses of childhood cancers.

**Methods:**

Medline (OVID), Embase (OVID) and PubMed were searched for studies published between January 2005 and December 2023, including children (<18 years) diagnosed with abdominal tumours, with no language restrictions. Pooled proportions of symptoms/signs were calculated. Sub-analyses were performed according to tumour location and age.

**Results:**

133 eligible studies were identified, totalling 8611 cases. The most frequently reported symptoms/signs were abdominal mass (39.3% (31.5% to 47.5%)), pain (14.5 (10.9% to 18.5%), abdominal swelling/distension (7.2% (3.3% to 12.1%)), haematuria (7.2% (2.9% to 6.2%)), fever (3.9% (2.2% to 5.9%)) and/or hypertension (2.6% (1.4% to 4.2%)).

For adrenal tumours, precocious puberty (20.6% (2.8% to 46.8%)), Cushing’s syndrome (16.4% (5.9% to 30.1%)) and/or hypertension (12% (2.8% to 25.3%)) were reported.

For liver tumours, abdominal mass (42.9% (0.0% to 100.0%)), abdomen mass and/or discomfort (16% (0.0% to 73.1%)), hepatomegaly (9.7% (0.0% to 60.7%)), abdominal swelling/distension (9.4% (0.0% to 64.0%)) and/or abdominal pain (7.7% (0.0% to 28.3%)) were reported.

For renal tumours, abdominal mass (49.7% (39.0% to 60.5%)), abdominal pain (12.3% (8.5% to 16.6%)), haematuria (10% (7.4% to 13.0%)), abdominal swelling/distension (5.4% (1.5% to 11.2%)), hypertension (4.7% (2.5% to 7.5%)) and/or fever (3.5% (1.9% to 5.5%)) were reported.

For neuroblastoma, abdominal mass (24% (7.0% to 46.4%), abdominal swelling/distension (9.2% (0.0% to 27.9%)), fever (7.4% (0.3% to 20.4%)), hepatomegaly (4.8% (0.0% to 19.8%)), anaemia/pallor (4.1% (0.0% to 13.3%)), abdominal pain (4% (0.0% to 13.4%)), screening/antenatal screening (3.4% (0.4% to 8.2%)) and/or opsoclonus-myoclonus-ataxia syndrome (2.7% (0.0% to 8.3%)) were reported.

**Conclusions:**

The clinical presentation of childhood abdominal tumours varies according to location and tumour type. These variations in presentation should be used to guide interventions to facilitate earlier diagnosis, such as the UK’s new Child Cancer Smart campaign.

**WHAT IS ALREADY KNOWN ABOUT THIS TOPIC**

- Childhood abdominal tumours pose a diagnostic challenge as evident in the variable time to diagnosis, up to 120 days.
- A larger size or later stage at time of diagnosis is associated with poorer outcomes for some abdominal tumours.
- The current literature of how these tumours present clinically is limited by bias from small sample sizes, single institution series and over-representation of rarer cases.

**WHAT THIS STUDY ADDS**

- This systematic review and meta-analysis presents a detailed appraisal of the current literature on the clinical presentation of abdominal tumours.
- These data are based on the largest sample of children diagnosed with abdominal tumours, from a wide range of different clinical contexts and countries, making its findings widely applicable.

**HOW THIS STUDY MIGHT AFFECT RESEARCH, PRACTICE OR POLICY**

- These data will be used to produce evidence-based guidelines for healthcare professionals and awareness tools for the public and healthcare professionals to aid prompt recognition of these signs/symptoms.

## Introduction

 Childhood cancer is estimated to affect 397 000 children globally each year.^1^ This impact varies significantly between countries, with approximately 3755 cases diagnosed per year in the UK.^2 3^ One in 194 males and 1 in 214 females are diagnosed with cancer before their 25th birthday.^2^

Abdominal tumours are a heterogeneous group of childhood cancers, encompassing renal and adrenal tumours, hepatoblastomas, abdominal neuroblastomas, abdominal lymphomas, gonadal germ cell tumours, abdominal rhabdomyosarcomas and carcinomas of the gastrointestinal tract.^4^ Individually, these make up a small proportion of childhood cancers; however, combined, they account for roughly 15% of all cases.^2^ With timely access to treatment, the 5-year survival estimate for childhood cancer is 81%.^2^ However, there is substantial variation in survival rates across abdominal tumour subtypes, from 41% for stomach and/or upper gastrointestinal cancer to 88% for renal tumours.^2^

Childhood abdominal tumours often present with non-specific symptoms/signs of an enlarging mass or features secondary to compression of nearby structures, which is plausibly related to size and stage at diagnosis, or symptoms due to high circulating hormones originating from functioning adrenal tissue.^5^,^9^ A high index of suspicion is therefore needed to recognise these infrequently encountered and sometimes complex presentations.

Time to diagnosis (TTD) of abdominal tumours can range from 6 to 25 days for renal tumours and up to 120 days for some gonadal germ cell tumours.^10^ This variable range in TTD is multifactorial^11^; however, one modifiable factor is an awareness of their clinical presentation.

Furthermore, there is evidence that Wilms tumours are significantly larger and at a more advanced stage at the time of diagnosis in the UK, which is associated with poorer survival outcomes compared with diagnosis at an earlier stage.^12^ A recent publication has also highlighted significant differences in proportions of children and young people (CYP) with metastases at diagnosis by country for neuroblastomas and Wilm’s tumours, with the UK having more CYP with metastases than France and Germany.^13^

Awareness campaigns have been shown to successfully reduce the TTD of childhood brain tumours.^14^ The Child Cancer Smart campaign aims to reduce the TTD of all childhood cancers by increasing awareness of the symptoms and/or signs.^15^ Earlier diagnosis of abdominal cancer may reduce exposure to more invasive therapy, potentially reducing treatment-related morbidity and mortality.

Current understanding of the clinical presentation of abdominal tumours is limited by small sample sizes,^16^,^20^ over-representation of rare subtypes of abdominal tumours,^21^,^26^ specifically selected cohorts,^27^,^29^ and/or bias towards cases with an advanced stage at diagnosis^30 31^ or the rarer, more unusual clinical presentations.^1732^,^37^ The tumour size and stage at diagnosis can vary between country-specific healthcare systems.^12 38^ Reported pre-diagnostic symptoms/signs may also vary by country, thus limiting the generalisability of findings from previously published single-institution series.

The aim of this study was to provide an evidence-based overview of the symptoms and/or signs of childhood abdominal tumours and explore how these vary according to anatomical location and age of diagnosis.

## Methods

### Search strategy and inclusion criteria

This review was conducted in alignment with Preferred Reporting Items for Systematic Reviews and Meta-Analysis and Strengthening the Reporting of Observational Studies in Epidemiology guidance.^39 40^

Our search strategy included the keywords: ‘abdominal tumour(s)’, ‘abdominal tumor(s)’, ’abdominal neoplasm(s)’, ‘wilm(s)’, ‘neuroblastoma(s)’, ‘diagnosis’, ‘signs’, ‘symptom(s)’, ‘signs and symptoms’, ‘presentation(s)’, ‘child’, ‘infant’, ‘adolescen(t)’, ‘Paediatric(s)’, and ‘pediatric(s)’. For full search terms and strategy, see online supplemental table S1.

Medline (OVID), Embase (OVID) and PubMed were searched for studies published from January 2005 to December 2023, with no language restrictions. All cross-sectional studies and case series, which included more than 10 paediatric cases (diagnosed under 18 years of age) with clinical presentation information, were included. Case reports or letters to the editor were excluded.

After removal of duplicate records, screening of titles, abstracts and full texts was conducted by two independent researchers (DS, LNC) and agreed with another researcher (J-FL). A comprehensive approach to identify all eligible grey literature was adopted, including searching reference lists and contacting authors.

### Data extraction

A standard extraction form was used by two independent researchers (DS, LNC), and quality was checked with other researchers (J-FL). Data items collected included study characteristics, year of publication, country, recruitment period, number of patients, study design, data source, tumour location and age at diagnosis. Clinical presentation data were recorded as reported. If the presence of a symptom/sign could not be ascertained, it was assumed to be absent. When it was not possible to separate symptoms reported in combination, they were extracted as symptom/sign clusters.

### Quality assessment

The quality of eligible studies was comprehensively assessed using a combination of criteria from Critical Appraisal Skills Programme (CASP) and Joanna Briggs Institute (JBI) qualitative assessment tools.^41 42^ The methodological domains evaluated include institution status, type of report, study population, sampling strategy, study design, case definition/verification and level of symptom detail reported (online supplemental table S2).

### Data analysis

The *metaprop* command^43^ in STATA V.18.5 (College Station, Texas, USA: StataCorp LLC)^44^ was used to estimate the pooled proportion. Given the anticipated high heterogeneity (I² >75%) across the eligible studies included in this review, a random-effects model (DerSimonian–Laird method) and the Freeman-Tukey double arcsine transformation were employed to calculate the pooled proportion (%) and 95% CIs for each symptom and sign. Heterogeneity was assessed using the I² statistic.

A predetermined threshold for symptoms/signs reported in 2% or more of the cohort was set, as a pragmatic compromise between identifying clinically relevant symptoms/signs and minimising the risk of overinterpreting non-specific symptoms/signs.

Sub-analyses were performed according to tumour locations and age at diagnosis.

## Results

**Figure 1:**
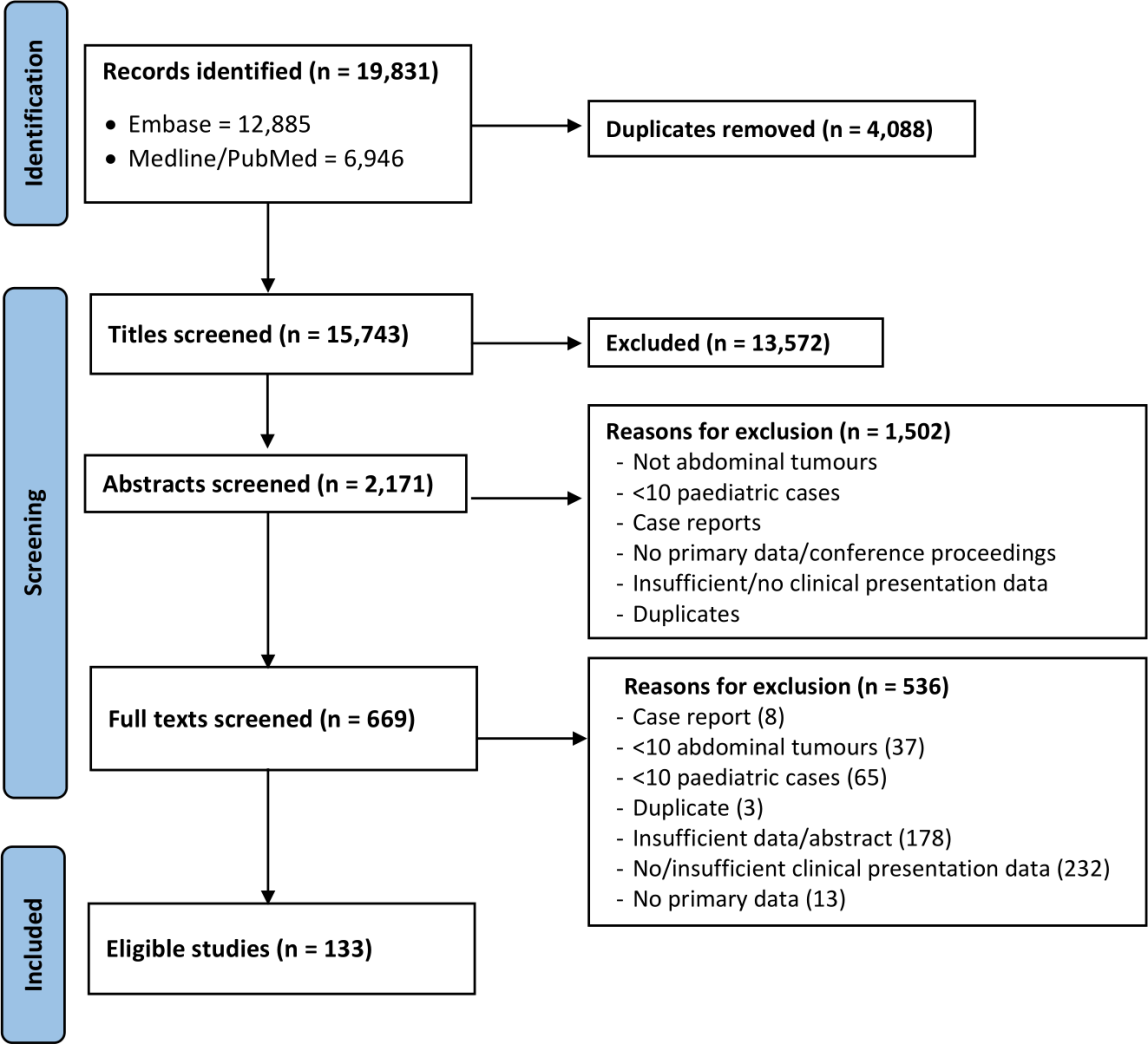
PRISMA (Preferred Reporting Items for Systematic Reviews and Meta-Analyses) flowchart of screening process to identify eligible studies.

A total of 19 831 studies were identified. After removal of duplicates, 15 743 studies remained. Screening of titles, abstracts and full texts identified 133 eligible studies (figure 1), including 8611 cases of childhood abdominal tumours, across 42 different countries. The characteristics and quality assessment of eligible studies can be found in online supplemental table S2.

In total, 220 combinations of symptoms/signs were extracted. Overlapping clinical features were clustered together into 147 symptoms/signs. Symptoms/signs were recorded as either pre-diagnostic or present at diagnosis in 94 studies, while 37 studies did not specify when the symptoms/signs were identified.

**Figure 2:**
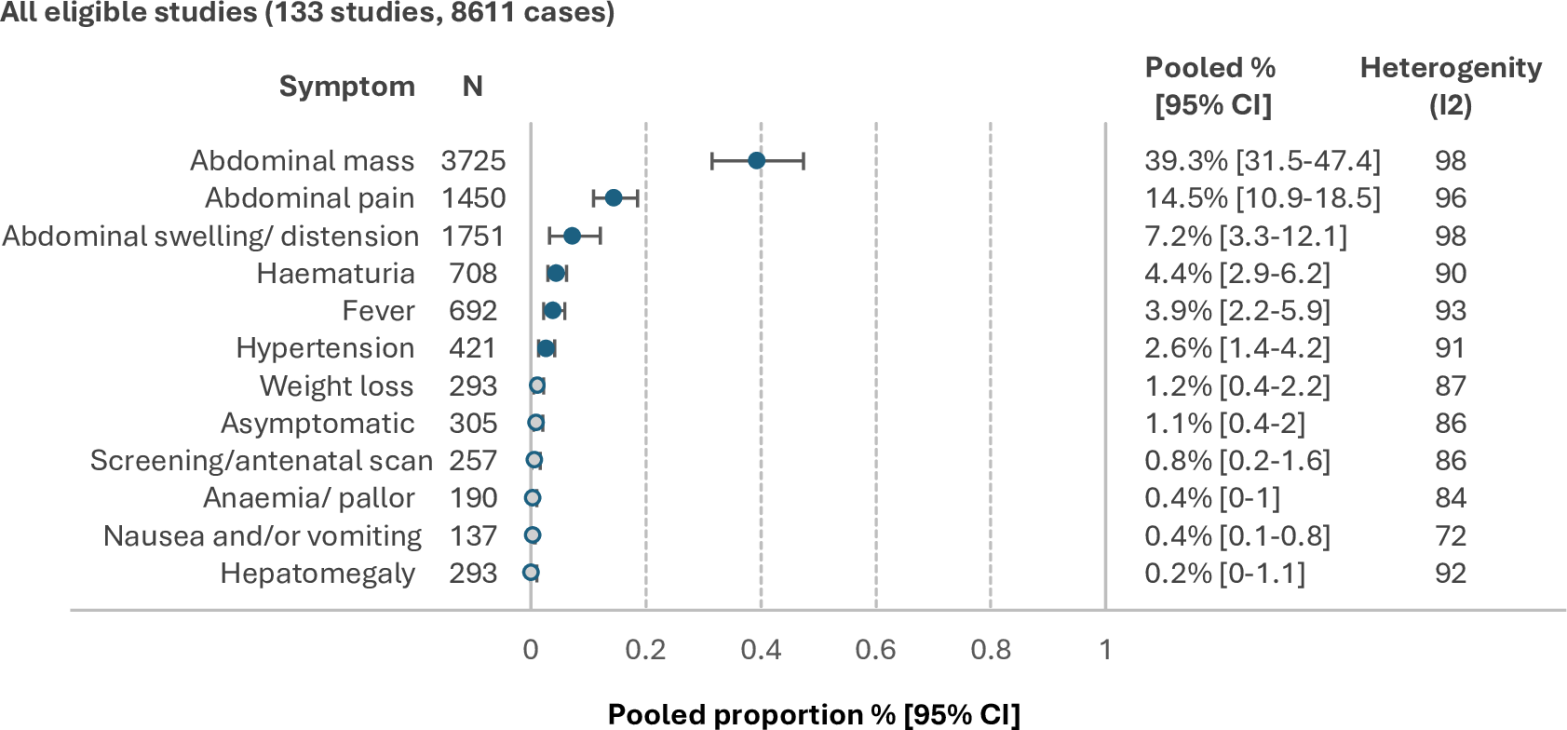
Pooled proportions for the most frequently reported pre-diagnostic symptoms/signs in the whole cohort. Hollow circles represent symptoms/signs with a pooled proportion of <2%.

Overall, the most common symptoms/signs reported include abdominal mass (39.3% (95% CI 31.5% to 47.5%)), abdominal pain (14.5 (10.9% to 18.5%)), abdominal swelling or distention (7.2% (3.3% to 12.1%)), haematuria (4.4% (2.9% to 6.2%)), fever (3.9% (2.2% to 5.9%)) and/or hypertension (2.6% (1.4% to 4.2%)) (figure 2).

### Tumour location

**Figure 3:**
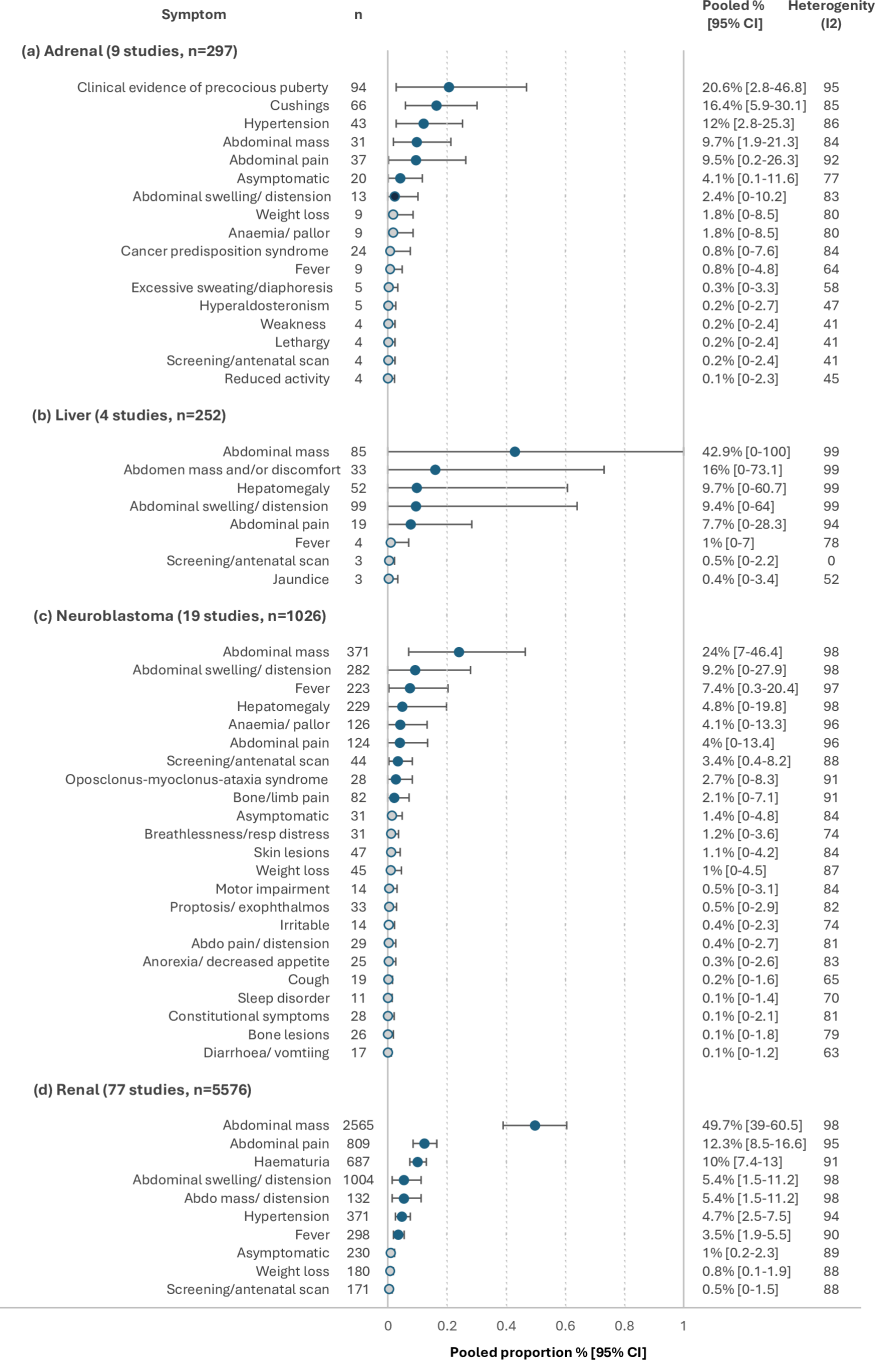
Pooled proportions for the most frequently reported pre-diagnostic symptoms/signs for abdominal tumours in (a) adrenal gland, (b) liver, (c) renal and (d) neuroblastoma. Hollow circles represent symptoms/signs with a pooled proportion of <2%.

Nine studies reported clinical presentation specific to adrenal tumours^2245^,^52^ (figure 3a). The most frequently reported symptoms/signs among these 297 cases were precocious puberty (20.6% (2.8% to 46.8%)), Cushing’s syndrome (16.4% (5.9% to 30.1%)), hypertension (12% (2.8% to 25.3%)), abdominal mass (9.7% (1.9% to 21.3%)), abdominal pain (9.5% (0.2% to 26.3%)) and/or asymptomatic (4.1% (0.1% to 11.6%)).

Four studies reported clinical presentation specific to liver tumours^53^,^56^ (figure 3b). The most frequently reported symptoms/signs among these 252 cases were abdominal mass (42.9% (0.0% to 100.0%)), abdomen mass and/or discomfort (16% (0.0% to 73.1%)), hepatomegaly (9.7% (0.0% to 60.7%)), abdominal swelling/distension (9.4% (0.0% to 64.0%)) and/or abdominal pain (7.7% (0.0% to 28.3%)).

Nineteen studies reported clinical presentation data specific to abdominal neuroblastoma^2831 33 35 36 57^,^70^ (figure 3c). The most frequently reported symptoms/signs reported among these 1026 cases included abdominal mass (24% (7.0 % to 46.4%)), abdominal swelling/distension (9.2% (0.0% to 27.9%)), fever (7.4% (0.3% to 20.4%)), hepatomegaly (4.8% (0.0% to 19.8%)), anaemia/pallor (4.1% (0.0% to 13.3%)), abdominal pain (4% (0.0% to 13.4%)), screening/antenatal screening (3.4% (0.4% to 8.2%)) and/or opsoclonus-myoclonus-ataxia syndrome (2.7% (0.0% to 8.3%)).

Seventy-seven studies reported clinical presentation data specific to renal tumours^2125 27 30 32 34 37 54 71^,^137^ (figure 3d). The most frequently reported symptoms/signs among these 5576 cases included abdominal mass (49.7% (39.0% to 60.5%)), abdominal pain (12.3% (8.5% to 16.6%)), haematuria (10% (7.4% to 13.0%)), abdominal swelling/distension (5.4% (1.5% to 11.2%)), hypertension (4.7% (2.5% to 7.5%)), fever (3.5% (1.9% to 5.5%)), asymptomatic (1% (0.2% to 2.3%)), weight loss (0.8% (0.1% to 1.9%)) and/or screening/antenatal scan (0.5% (0.0% to 1.5%)).

### Age of diagnosis

**Figure 4:**
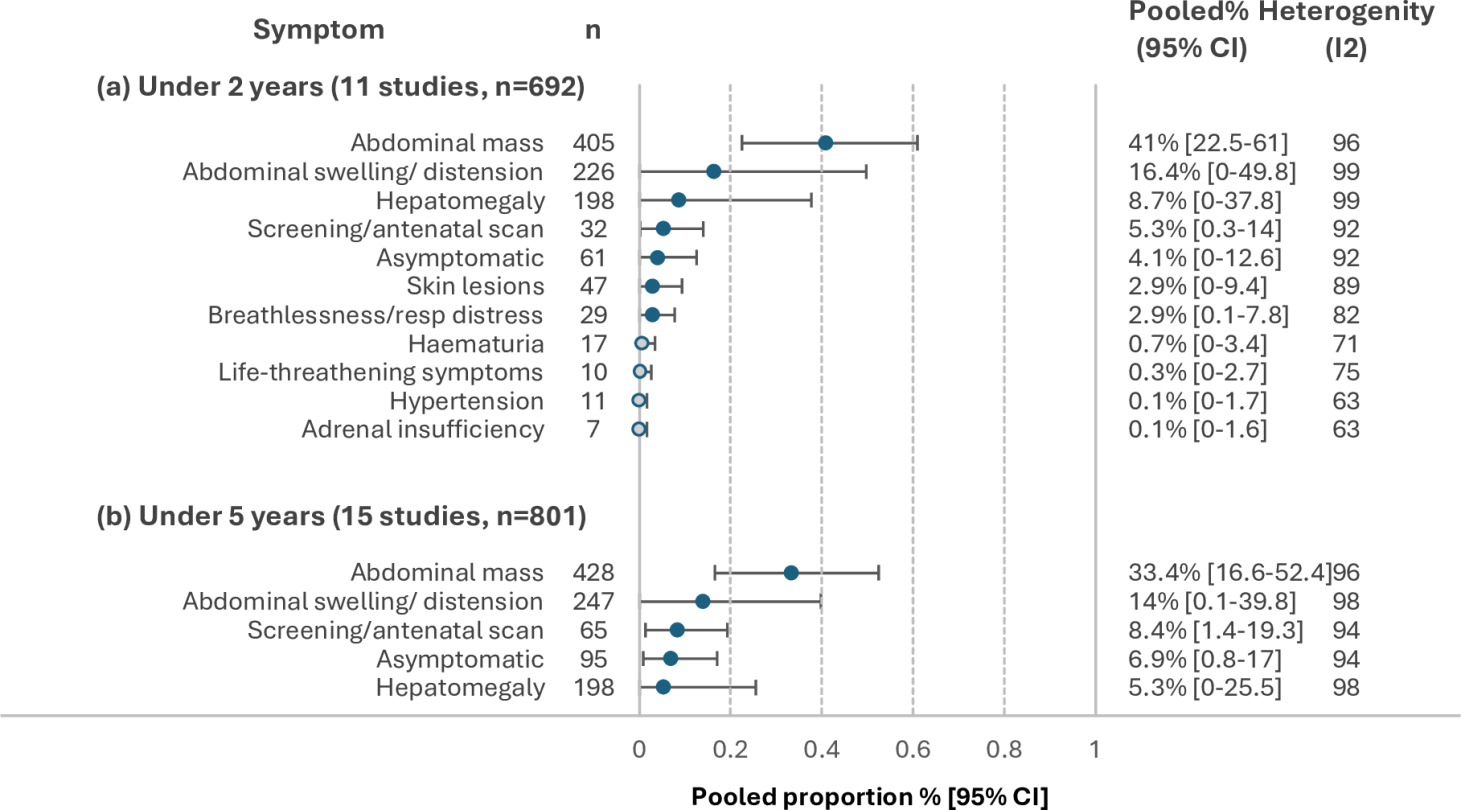
Pooled proportions for the most frequently reported pre-diagnostic symptoms/signs for abdominal tumours diagnosed in (a) under 2 years of age and (b) under 5 years of age. Hollow circles repesent symptoms/signs with pooled proportions of <2%.

Eleven studies reported clinical presentation data specific to diagnosis under 2 years of age^27^,^2931 65 66 69 97 110 138^ (figure 4a). The most frequently reported symptoms/signs among these 692 cases included abdominal mass (41% (22.5% to 61.0%)), abdominal swelling/distension (16.4% (0.0% to 49.8%)), hepatomegaly (8.7% (0.0% to 37.8%)), screening/antenatal scan (5.3% (0.3% to 14.0%)), asymptomatic (4.1% (0.0% to 12.6%)), skin lesions (2.9% (0.0% to 9.4%)) and/or breathlessness/respiratory distress (2.9% (0.1% to 7.8%)).

Fifteen studies reported clinical presentation data specific to diagnosis under 5 years of age^27^,^2931 35 65 66 69 97 103 106 110 127 138^ (figure 4b). The most frequently reported symptoms/signs among these 801 cases included abdominal mass (33.4% (16.6% to 52.5%)), abdominal swelling/distension (14% (0.1% to 39.8%)), antenatal scan (8.4% (1.4%% to 19.3%)), asymptomatic (6.9% (0.8% to 17.0%)) and/or hepatomegaly (5.3% (0.0% to 25.5%)).

## Discussion

This extensive review, including 8611 cases, has identified that the most common symptoms/signs at diagnosis with any childhood abdominal cancer are abdominal mass, swelling and distension with or without pain, reflecting previously published, smaller cohort data.^1618^,^20^

The recently published Childhood Cancer Diagnosis study showed that the median total diagnostic interval for abdominal tumours had high variability; 2.3 weeks for renal tumours, 4.4 weeks for neuroblastoma, 5.1 weeks for liver tumours and 5.9 weeks for germ cell tumours.^139^ Even for those with shorter intervals, the UK has larger tumour volumes and greater cases with metastases than its European counterparts for Wilm’s tumours and neuroblastoma, which are two of the most common abdominal tumours in CYP.^12 13^ There is therefore an urgent need to prioritise early diagnosis for this particular group, given that survival rates for these tumour types are worse than in other cancers, such as leukaemia.^140^ The HeadSmart campaign is an example of a successful early diagnosis intervention, where increasing awareness was associated with halving the TTD for CYP with brain tumours from a median of 14.4 weeks to 6.7 weeks, 5 years post launch.^14^ We hypothesise that using these data to raise awareness of the presentation of abdominal tumours could accelerate diagnosis in the same way.

### Tumour location

The symptoms/signs of abdominal tumours vary according to tumour location and the tissue of origin.^15 141 142^

Alongside an abdominal mass, swelling/distension or pain, haematuria and hypertension are

frequently reported in childhood renal tumours. The high frequency of haematuria and hypertension reported in the combined sample likely reflects the large proportion of studies focusing on renal tumours, 77 of 133 studies included. Previous smaller studies looking at specific cohorts of renal tumour subtypes reported variable frequency of these symptoms/signs.^21 143 144^ However, when all subtypes are combined, the most frequent symptoms/signs are similar to those identified in this analysis.^95 128^ Increasing awareness of haematuria and hypertension in association with renal tumours will encourage a higher index of suspicion of cancer and identify those who warrant further investigation.

Precocious puberty was reported in 94 of 297 cases with adrenal tumours, defined as a combination of virilisation, hirsutism, breast, penile or testis enlargement, bilateral gynaecomastia, increase in pubic hair, deepening voice and/or acne. Adrenal tumours presenting with precocious puberty are extensively published in the literature as case reports and small case series.^145^,^149^ The frequency of precocious puberty, Cushing’s syndrome and hyperaldosteronism in these results reflects previous literature showing a high frequency of hormonally functioning adrenal tumours.^150^ Cancer predisposition syndromes were frequently reported in studies exploring cases with adrenal tumours.^150^ This association between predisposition syndromes and childhood cancer is also applicable to the other abdominal childhood tumours, including renal tumours and neuroblastomas, which is clinically relevant to the argument for more research into surveillance and screening for childhood cancer.^151^

Neuroblastomas most frequently occur in the abdomen, with the thoracic region being the second most common location.^152^ Of the included studies, eight reported symptoms in a combined cohort of abdominal, pelvic, thoracic and cervical locations which could not be separated.^2935 36 60 63 66 68^,^70^ In addition to the wide range of non-specific symptoms/signs, including fever and anaemia, among neuroblastoma cases, the more specific presentation with opsoclonus-myoclonus syndrome was reported in 28 of 1026 cases. Oposclonus-myoclonus syndrome is a neurological finding that is often associated with neuroblastomas as a paraneoplastic phenomenon. It is important to raise awareness of this relatively frequent presenting feature and its association with underlying childhood neuroblastomas in the locations cited above, as it is often under-reported in small cohorts in previous literature.^152^

Among liver tumours in this analysis, jaundice is reported in very few cases, reflecting previous studies.^153 154^ This may be because jaundice develops at a more advanced stage of malignancy when liver function has become impaired secondary to tumour infiltration. Ideally, liver tumours would be diagnosed before functional impairment develops.

### Age at diagnosis

Tumour location varies according to age, reflecting age-related spurts in growth velocities of certain tissues; therefore, different types of tumours are relatively more common at different ages.^155^ For example, the majority of neuroblastomas, renal, adrenal and liver tumours present before the age of 5 years.^2^ In comparison, there is a bimodal distribution of gonadal germ cell tumours, with highest incidence rate aged under 4 years and over 15 years.^2^ While gastrointestinal tract tumours are relatively more frequent in older children and adolescents, it is also important to highlight the higher proportions of asymptomatic presentations reported in the under five age group. The reason for this is unknown but likely due, in part, to differences in international practices for child health surveillance and physical examination in early years.

As clinical presentation differs according to tumour location and given the age-related relationship with tumour location, it would be important to incorporate these relevant findings into raising awareness interventions by emphasising the relevant symptoms/signs according to age. For example, breathlessness or respiratory distress is frequently reported in those aged <2 years, which is likely due to the mass effect from an enlarging abdominal mass splinting the diaphragm within a relatively smaller abdominal cavity. Unfortunately, insufficient detail in reporting symptoms/signs according to age among the included studies meant there was not enough data to compare age-related variations in clinical presentation. Further studies to explore age-related differences in clinical presentation are warranted. This is particularly true for older children and adolescents, who are at increased risk of the more rarely encountered abdominal tumour locations and a prolonged TTD.^156^

### Strengths and limitations

To the best of our knowledge, this review provides an overview of presenting symptoms/signs in the largest cohort of childhood abdominal tumours yet reported. The extensive, systematic approach taken to identify all eligible literature provides support for the reliability of these findings. The studies included are from a wide range of countries, supporting the generalisability and applicability of these findings to various clinical contexts.

These results are limited by the quality of symptom/sign detail and the reporting of combined symptoms/signs which could not easily be extracted from studies. The results are limited by our assumption that if a symptom/sign was not reported, it was not present and may therefore be under-represented in the results, especially in those less common symptoms.

The high heterogeneity for results reflects the substantial variation across studies of variable sample sizes and characteristics. Therefore, it is important not to overinterpret the absolute pooled proportions or their rank. Instead, the emphasis is on the nature of symptoms/signs reported and how these vary according to location and age.

### Implications of findings

This extensive evidence-based overview enhances our current understanding of the clinical presentation of childhood abdominal tumours. These data have been used in a Delphi consensus process to develop statements for inclusion in a new clinical guideline.^157^ The guideline will be published shortly and translated into public and professional facing awareness messages, as part of the recently launched Child Cancer Smart campaign.^158^

This campaign aims to accelerate diagnosis for all childhood cancers by providing clear evidence-based guidance to both the public and professionals to support prompt recognition, assessment and investigation, using the same model as the award-winning HeadSmart campaign.^14^ The campaign was launched as part of Childhood Cancer Awareness month in September 2025 in order to deliver immediate impacts to children and families based on the outcomes of the systematic reviews and guidelines developed in the earlier part of the programme.^158^

## Conclusion

Childhood abdominal tumours are challenging to diagnose early. This study provides an overview of clinical presentations of childhood abdominal tumours and highlights how presentation differs according to tumour location, which varies with age. These results are being used to inform a new clinical guideline for healthcare professionals and for public awareness through the new Child Cancer Smart campaign, aiming to accelerate diagnosis of childhood abdominal tumours.

## Supplementary material

10.1136/archdischild-2025-329097 online supplemental table 1

10.1136/archdischild-2025-329097 online supplemental table 2

## Data Availability

Data are available upon reasonable request.
