# Supplementary material for: Spotting childhood abdominal tumours: a systematic review and meta-analysis of the clinical presentation
Source: Arch Dis Child. 2025 Oct 5;111(2):e329097. doi: 10.1136/archdischild-2025-329097 (PMC12911601; doi:10.1136/archdischild-2025-329097)
Supplement: online supplemental table 1 [file archdischild-111-2-s001.pdf]

Table S1 Literature review search terms and strategy

1	abdominal tumour*.ti,ab.
2	abdominal tumor*.ti,ab.
3	abdominal neoplasm*.ti,ab.
4	Wilms*.ti,ab.
5	Neuroblastoma*.ti,ab.
6	exp Abdominal Neoplasms/
7	Abdominal neoplasms/di
8	Wilms/di [Diagnosis]
9	Neuroblastoma/di [Diagnosis]
10	or/1-9
11	diagnosis.ti,ab.
12	exp Diagnosis/
13	sign*.ti,ab.
14	symptom*.ti,ab.
15	exp "Signs and Symptoms"/
16	(signs and symptoms).ti,ab.
17	presentation*.ti,ab.
18	diagnos*.ti,ab.
19	or/11-18
20	10 and 19
21	limit 20 to human
22	limit 21 to ("all infant (birth to 23 months)" or "all child (0 to 18 years)" or "newborn infant (birth to 1 month)" or "infant (1 to 23 months)" or "preschool child (2 to 5 years)" or "child (6 to 12 years)" or "adolescent (13 to 18 years)")
23	limit 22 to (infant <to one year> or child <unspecified age> or preschool child <1 to 6 years> or school child <7 to 12 years> or adolescent <13 to 17 years>)
