# Supplementary material for: Spotting childhood abdominal tumours: a systematic review and meta-analysis of the clinical presentation
Source: Arch Dis Child. 2025 Oct 5;111(2):e329097. doi: 10.1136/archdischild-2025-329097 (PMC12911601; doi:10.1136/archdischild-2025-329097)
Supplement: online supplemental table 2 [file archdischild-111-2-s002.pdf]

Table S2 Characteristics of all articles included in the analysis and quality assessment

ID	Authors	Year	Country	n	mean age	median age	age range	presenting symptom/ prior to diagnosis	Institution status	Type of report	Study population	Sampling strategy	Study design*	Case definition and verification	Level of detail reported (no. of symptoms)
1	User et al.	2015	Turkey	22	-	2.5y	2m - 7 y	y	tertiary	full article	all children diagnosed/treated at hospital between 1980-2013	inclusive, clear	R	NS	>4
2	Fadoo et al.	2009	Pakistan	23	-	4y	1-9 yrs	y	tertiary	full article	all children diagnosed/treated at hospital between 1980-2013	clear exclusion	R	y	>4
3	Lin et al.	2017	China	48	-	-	1 day - 14 yrs	y	tertiary	full article	all children treated operatively at institution between 2001-2015	inclusive, exclusion criteria NS	R	y	>4
4	Anwar et al.	2017	Pakistan	175	3y	3.5y	NS paediatrics	NS	tertiary	full article	all children diagnosed with wilms tumour between 2009-2013	inclusive, exclusion criteria ns	R	y	1
5	D'Angelo et al.	2017	Italy	124	12.8m	-	<24 m	y	tertiary (>4)	full article	all children registered on national registry diagnosed with wilms tumour between 2003-2010	restrictive	R	n	3
6	Elashry	2012	Egypt	25	34.5m	-	NS paediatrics	y	Tertiary	full article	all children registered at institution between 1993 and 2009 with bilateral disease	restrictive	R	NS	>4
7	Ritchey et al.	2008	United States	45	33m	-	NS paediatrics	y	teriary (1)	full article	all children enrolled in trials between 1979 and 2003 with ureteral extension of wilms tumour	restrictive	P	NS	>4
8	Guruprasad et al.	2013	India	81	3.3y	-	1-7 yrs	y	NS (1)	full article	all children diagnosed at institution between 2003 and 2010	inclusive, clear	R	NS	4
9	Hadley et al.	2013	South Africa	20	3.6y	2.5y	10 m - 9 y	y	NS (1)	full article	all children diagnosed with bilateral disease	restrictive	R	NS	>4
10	Hadley & Sheik-G	2010	South Africa	14	46m	-	14 m - 12 y	y	NS (1)	full article	all children diagnosed with CCSK in centre between 1990-2008	restrictive, unclear of exclusion criteria	R	NS	1
11	Chan et al.	2010	Hong Kong	13	-	37m	9 m - 10 y	y	tertiary (1)	full article	all children who underwent nephrectomy for WT between 1996-2011	restrictive exclusion criteria	R	NS	3
12	Illade et al.	2018	Spain	40	-	2.5y	4 m - 15 y	y	NS (1)	full article	all children diagnosed with nephroblastoma between 2002-2016	inclusive, exclusion criteria	R	NS	>4
13	Provenzi et al.	2014	Brazil	45	43.9m	-	4 m - 101 m	y	tertiary (1)	full article	all children diagnosed with WT between 1989-2009	unclear inclusion criteria	R	NS	>4
14	Rias et al.	2016	Morocca	52	-	4y	17 m - 14 y	NS	tertiary (1)	full article	all children treated for WT at institution between 2005-2010	unclear inclusion criteria	R	NS	4
15	Atanda et al.	2015	Nigeria	30	4.8y	-	NS paediatrics	y	tertiary (1)	full article	all cases diagnosed with WT at institution between 2009-2013	clear exclusion criteria	NS	y	4
16	Sah et al.	2010	Nepal	60	36m	-	1m-13y	y	tertiary (1)	full article	all cases diagnosed with wilms tumor between 1998-2008	exclusion criteria clear	R	y	4
17	Zugor et al.	2010	Germany	65	-	-	NS paediatrics	NS	tertiary (1)	full article	all children treated for WT between 1965-2004	NS	NS	y	
18	Ali et al.	2011	Egypt	35	-	6y	2.5-15 yrs	y	Tertiary (1)	full article	all children treated for abdominal NHL between 2005-2010	exclusion criteria clear	P	y	>4
19	Fumino et al.	2016	Japan	14	-	5.5m	0-64 m	NS	tertiary (1)	full article	all children treated with radiotherapy at institution between 1989 - 2015	inclusion criteria, restrictive	R	NS	2
20	Kostyrka et al.	2011	Australia	12	-	-	<28 days	y	NS (2)	full article	all children diagnosed with neuroblastoma between 1999-2009	restrictive, clear inclusion criteria	NS	NS	2
21	Rathore et al.	2016	India	112	15.3y	-	1.5 - 20 yrs	y	tertiary (1)	full article	all children diagnosed with ovarian cancer between 1990-2014 at institution	inclusive, exclusion criteria nS	R	NS	>4
22	Salim et al.	2011	United Kingdom	91	-	1.9y	0-14.9y	NS	tertiary (1)	full article	all children treated for neuroblastoma between 1985-2005	inclusive exclusion criteria NS	R	y	>4

ID	Authors	Year	Country	n	mean age	median age	age range	presenting symptom/ prior to diagnosis	Institution status	Type of report	Study population	Sampling strategy	Study design*	Case definition and verification	Level of detail reported (no. of symptoms)
23	Bansal et al.	2007	India	103	41.5m	-	12.5 m - 12.5 y	y	NS (1)	full article	all children treated for neuroblastoma between 1190-2004 for aged >1 year at diagnosis	exclusion criteria NS	R	y	>4
24	Aldaqal et al.	2013	Saudi Arabia, Egypt	46	4.5y	-	1.5-6.5 y	y	tertiary (2)	full article	all children diagnosed with abdominal neuroblastoma between 2008-2012	exclusion criteria NS	R	y	>4
25	Yao et al.	2012	China	67	39m	-	2.4-168 m	y	tertiary (1)	full article	all children diagnosed with Wilms tumour between 1998 -2008.	exclusion criteria NS	R	y	>4
26	Hadley and Heerden	2017	South Africa	45	2.8y	1.9y	4d-11y	y	tertiary (1)	full article	all children diagnosed with neuroblastoma and received surgical treatment between 2001-2012	exclusion criteria clear	R	y	>4
27	Atteby et al.	2012	Cote d'Ivoire	66	7.2y	-	5m-15y	NS	tertiary (1)	full article	all children diagnosed with a liver tumours between 1991-2007	exclusion criteria NS	R	NS	3
28	Koh et al.	2015	Singapore	27	-	-	<1 y	y	tertiary (1)	full article	all children diagnosed with neuroblastoma under 12 months between 1998-2012	exclusion criteria clear, restrictive	R	NS	>4
29	Zhang et al.	2016	china	54	6.9y	-	5y-15y6m	y	tertiary (1)	full article	children aged 5 years and older diagnosed with NB and admitted to our hospital between February 2006 and January 2016	exclusion criteria ns, restrictive	R	y	>4
30	Chang et al.	2012	china	95	3.2y	3.2y	<18y	y	tertiary (1)	full article	all children admitted with nephroblastome between 2001-2007	inclusion criteria, no exclusion criteri	R	y	4
31	Chan et al.	2014	Hong Kong	76	3.2y	-	0.3-14.7 y	y	tertiary (5)	full article	all children diagnosed and treated for kidney tumours in Hong Kong between 1990-2010		P	y	>4
32	Erginel et al.	2014	Turkey	71	3.1y	-	2d-7y	y	tertiary (1)	full article	children with wilms tumour diagnosed and treated between 1990-2014	exclusion criteria NS	R	y	>4
33	Kaplan et al.	2013	Turkey	20	-	-	<19 y	y	tertiary (1)	full article	children diagnosed with colorectal cancer between 2003-2010	exclusion criteria NS	R	NS	>4
34	Li et al.	2012	China	98	-	48m	12d-156m	NS	tertiary (1)	full article	all children newly diagnosed neuroblastoma between 2000-2006	inclusion criteria clear	R	y	>4
35	Collins et al.	2009	Belgium	31	4.4y	-	0-15.2y	y	NS (1)	full article	all children diagnosed with renal tumours between 1990-2009	exclusion criteria clear	R	NS	>4
36	Chan et al.	2014	Singapore	21		3.3y	0.25-10.8 y	y	tertiary (1)	abstract	all children with malignant renal tumours between 1997-2012	exclusion criteria NS	R	NS	>4
37	KTS et al.	2015	Singapore	29	-	3.5y	0.25y-11y	y	tertiary (1)	abstract	all children diagnosed with malignant renal tumours between 1997 and 2012	inclusion criteria clear. exclusionc crtieria NS.	R	y	>4
38	Eke	2015	Nigeria	31	-	-	<18 y	y	tertiary (1)	abstract	all children admitted with wilms tumour between 2011-2014	exclusion criteria NS	NS	NS	2
39	Fitzgerald et al.	2016	Tanzania	28	-	-	<18 y	y	tertiary (1)	abstract	all children admitted with wimls tumour between 2008-2009	exclusion criteria NS	NS	NS	4
40	Kashari et al .	2015	Saudi Arabia	27	3.4y	-	NS paediatrics	y	tertiary (1)	abstract	all children with wilms tumour between 2000-2013	exclusion criteria NS	R	y	2
41	Okur et al.	2015	Turkey	406	5.4y	-	10 d-17y	y	NS	abstract	all children with intraabdominal malignant tumour diagnosed between 1991-2015	exclusion criteria NS	R	NS	>4
42	Okur et al.	2014	Turkey	56	50.7m	-	8-204 m	NS	NS	abstract	all children with wilms tumour diagnosed between 1991-2014	exclusion criteria NS	R	NS	>4
43	Owens et al.	2010	France	187	-	2.8y	2d - 21 y	y	tertiary (1)	abstract	all children presented with a renal tumour between 2000-2009	exclusion criteria NS	R	not all	>4
44	Panagopoulou et al.	2010	Greece	56	-	-	8m-12 y	y	tertiary (1)	abstract	all children with wilms tumour between 1993-2008	exclusion criteria NS	R	not all	>4
45	Ranalli et al.	2012	United States	83	6.3m	7m	<12 m	y	NS (1)	abstract	all children presented with renal tumours between 1945-2011	exclusion criteria NS	R	y	3

ID	Authors	Year	Country	n	mean age	median age	age range	presenting symptom/ prior to diagnosis	Institution status	Type of report	Study population	Sampling strategy	Study design*	Case definition and verification	Level of detail reported (no. of symptoms)
46	Sitthi-amorn et al.	2013	United States	39	4.2y	-	0-19.6y	y	NS (1)	abstract	all children diagnosed with wilms tumour between 1995-2007	exclusion criteria NS	R	NS	4
47	Wee Sim et al.	2010	Singapore	23	-	-	<18 y	y	NS (1)	abstract	all children surgical treatment for malignant renal tumours between 1997-2009	exclusion criteria NS	R	y	>4
48	Chai et al.	2009	China	11	37m	-	<18 y	NS	NS (1)	abstract	all children diagnosed with neuroblastoma between 2001-2008	exclusion criteria NS	NS	y	4
49	Haart Isaacs Jr	2008	USA	210	-	-	<2m	NS	tertiary (2)	full article	all children diagnosed with renal tumours aged <2 months old between 1960 and 2007	restrictive. inclusion and exclusion criteria clear	R	y	>4
50	Axt et al.	2013	Kenya	133	-	-	<15 y	NS	NS (4)	abstract	all children treated for wilm tumours between 2008-2012	exclusion criteria NS	NS	Y	4
51	D'Angela et al.	2017	Italy	117	12.8m	13m	<24 months	NS	tertiary	full article	all aged <2 years diagnosed with wt	all registered on specific treatment protocol	R	Central review of 80%	2
52	Alakaloko et al	2022	Nigeria	40	3.9y	-	6 m-12 y	y	Tertiary	full article	all treated for Wilms tumour over 5 year period	all-inclusive over 5 year period	R	y	>4
53	Tripathy et al.	2020	India	63	-	-	7m-6y	y	tertiary	full article	aged <14years with intra-abdominal tumour arising from adrenal/kidney who was operated on	all-inclusive over 5 year period	R	y	>4
54	Kuhlen et al.	2022	Germany	14	5.7y	4.7y	0.2y-15.8y	y	tertiary	full article	Initially misdiagnosed as neuroblastoma	all-inclusive from 1997-2019	R	y	>4
55	Singh et al.	2022	India	26	-	10.5m	10day-14.8y	NS	NS (1)	abstract	retro-peritoneal germ cell tumours in one hospital from 1998-2022	all-inclusive	R	y	3
56	Andreeve et al.	2020	Russia	29	-	1.9m	0.2m-12.6m	y	tertiary	full article	all treated at single centre for bilateral adrenal neuroblastoma from 2012-2018		R	n	>4
57	Montalto et al.	2022	Italy	210	-	-	0-1y	NS	NS (>1)	full article	aged 0-18years diagnosed with stage 4 adrenal neuroblastoma on national registry between 1971 and 2016.	consecutive	R	y	>4
58	Van Peer et al.	2021	The Netherlands	24	-	-	7-59m	y	tertiary	full article	aged <19years bilateral renal tumours	all	R	y	>4
59	Solomon et al.	2021	South Africa	18	-	30m	9-145m	NS	NS (2)	full article	aged 0-16years with bilateral Wilm's tumour from 2003 -2013		R	y	>4
60	Veerabadha et al.	2018	India	21	6y	-	3-14y	y	tertiary (1)	full article	all treated at tertiary centre from 2010-2016	inclusive	R	y	>4
61	Erburu et al.	2020	Spain	27	-	-	<18m	NS	NS	full article	all treated at single centre for between 2009- 2019		R	y	>4
62	Roy et al.	2022	The Netherlands	163	-	35m	0-226m	y	tertiary (1)	full article	all diagnosed with a renal mass between 2015-2019		NS	y	3
63	Hol et al.	2020	International - Europe	39	-	22m	6-44m	y	NS (>1)	full article	included all pt on x2 international registries		R	y	2
64	Wang et al.	2019	China	28	5.3y	-	10m-13y	y	NS	full article	Included all patients treated for adrenocotical tumour between 2010-2017		R	y	>4
65	Lin et al.	2018	China	26	-	1.2y	NS paediatrics	y	NS	full article	Included all patients treated for adrenocotical tumour between 2010-2017		R	y	3
66	Fang et al.	2020	China	63		30.5m	1-120m	y	NS	full article	included all patients presenting to institute with neuroblastoma between 2016-2018		R	y	2
67	Yadav et al.	2018	India	13	1.5m	-	<9y	NS	NS	abstract	Unclear	NS	P	NS	2

ID	Authors	Year	Country	n	mean age	median age	age range	presenting symptom/ prior to diagnosis	Institution status	Type of report	Study population	Sampling strategy	Study design*	Case definition and verification	Level of detail reported (no. of symptoms)
68	Castillo et al.	2021	Mexico	34	11.7y	-	NS paediatrics	NS	Tertiary	abstract	review of clinical cases <18 years with GCT ovary	NS	R	NS	
69	Miele et al.	2020	Italy	13	-	17m	0-82m	y	Tertiary	full article	adrenocortical tumour aged <18 years	cohort study	R	y	>4
70	Joseph et al.	2021	India	50	3.4y	2.5y	3m-14y	NS	tertiary	full article	aged <15 years intra-abdominal tumors	all included	R	y	>4
71	Alharti et al.	2022	Saudi Arabia	37	-	29m	4-99m	y	tertiary	abstract	Paediatric patients with wilm tumour treated at single centre between 2004-2016	NS	R	NS	>4
72	Kaplan et al.	2019	Turkey	32	-	18y	10-19y	y	multi-centre	full article	aged 10-19years with colorectal cancer treated at centres between 2003-2015	NS	R	NS	>4
73	Hasbay et al.	2022	Turkey	22	-	-	1d-16y	y	NS	full article	aged 0-18y treated for sacrococygeal germ cell teratoma	Search hospital system	R	y	>4
74	Salih et al.	2021	Sudan	51	3.4y	-	0.5-10y	y	NS	full article	aged <16y diagnosed for wilms tumour at centre between 2006-2010	NS	R	y	>4
75	Ma et al.	2017	China	13	5.4m	-	0.25-18m	NS	tertiary	full article	all patients treated at centre between 2004-2017 with diagnosed confirmed histopathology	NS	R	y	3
76	Koh et al.	2022	Korea	439		27.1m	0-225.5m	y	multicentre (26)	full article	2001-2015 all patients newly diagnosed renal tumours	comprehensively reported - reason for exclusions	R	20 not confirmed by pathological diagnosis	3
77	Citak et al.	2018	Turkey	48	53.3m	-	1-192m	NS	NS	abstract	2008-2017 children with renal tumours	NS	R	NS	3
78	Picard et al.	2019	France	95	5y	-	0-17y	y	multi-centre (23)	full article	all diagnosed with adrenal neoplasm positive pathology between 2000-2018	reported reasons for exclusion and missing data	R	y	>4
79	Virgone et al.	2020	Italy	15	152.4m	-	70-200m	y	multicentre (11)	full article	all diagnosed with neuroendocrine tumours on registry between 2000-2020	clear inclusion/exclusion criteria	R	NS	>4
80	Otham et al.	2021	Singapore	20	-	9.8y	1.5-15.3y	y	NS	full article	All diagnosed with gastrointestinal lymphoma operated on between 2000-2019	clear inclusion/exclusion criteria	R	y	2
81	Monneraye et al.	2019	France	317	4y	-	0-17.6y	NS	NS	full article	All diagnosed/treated for renal tumour <18years between 1993-2015	clear inclusion/exclusion criteria	R	y	2
82	Qureshi et al.	2021	India	43	-	4.5y	1.6-13.5y	NS	NS	full article	all wilms tumour thrombus with wilms tumour	Exclusion criteria clear	P	y	3
83	Varela et al.	2019	Argentina	28	-	5.2y	2m-18y	y	NS	abstract	all adrenal tumours operated on using laparoscopic approach between 2003-2018		R	y	1
84	Parada-Avendano et al	2023	Spain	166	-	paediatric NS	<18y	y	y	full article	all <18years diagnosed with wilms tumour or hepatoblastoma between 1997-2021	exclusion criteria clear	R	NS	3
85	Fiori et al.	2018	Brazil	21	-	-	NS paediatrics	NS	NS	abstract	case series treated for hepatic tumours between 2004-2016	NS	R	NS	3
86	Asfour et al.	2020	Egypt	92	-	3y	1m-9y	y	NS	full article	patients with unilateral wilms tumour treated at centre between 2008-2017	exclusion criteria clear	R	y	3
87	Sun et al	2022	China	14	-	20m	13-54m	y	tertiary	full article	patients with oposclonus-myoclonus associated with neuroblastoma between 2011-2019	exclusion criteria clear	R	y	4
88	Mohajerzadeh et al.	2021	Iran	115	-	-	NS paediatrics	y	NS	full article	patients surgical treatment wilms tumour between 1992-2016	exclusion criteria clear	R	y	>4

ID	Authors	Year	Country	n	mean age	median age	age range	presenting symptom/ prior to diagnosis	Institution status	Type of report	Study population	Sampling strategy	Study design*	Case definition and verification	Level of detail reported (no. of symptoms)
89	Faizen et al.	2018	Pakistan	191	-	-	0-16y	y	NS. multicentre (2)	full article	patients treated for abdominal tumour in 2016 year	exclusion criteria clear	R	y	>4
90	Elayadi et al.	2020	Egypt	24	5.3y	-	2-12.7y	y	NS	full article	patients treated for metastatic wilms tumour	exclusion criteria clear	R	NS	3
91	Anand et al.	2023	India	22	-	2.5m	1.6-3.8m	y	NS	full article	patients treated for stage 4s neuroblastoma at single institute	exclusion crieteria clear	R	All except 1 case confirmed histopahtological diagnosis	4
92	Faraj et al	2021	Iraq	119	-	32m	2-155m	NS	NS	abstract	Patients admitted for treatment of newly diagnosed NB	NS	R	NS	3
93	Hadousa et al	2018	Tunisia	15	3.9y	-	<18y	y	NS	abstract	patients diagnosed with stage 4 wilms tumours	NS	R	NS	3
94	Sachdeva et al.	2019	India	61	-	2.5y	18d-11.6y	y	NS	abstract	patients diagnosed with Wilms tumour between 2005-2018	exclusion clearly reported	R	NS	2
95	Chabchoub et al.	2022	Tunisia	22	4y	-	8m-8y	NS	NS	abstract	patients treated for metastatic nephroblastoma between 1994-2020	NS	R	NS	1
96	Vermesche et al	2020	France	14	12.3y	-	2.2-17.5y	y	NS, multicentre	full article	paediatric patients diagnosed with peritoneal mesothelioma	Inclusion criteria clearly reported	R	y	>4
97	Shyirambere et al.	2022	Rwanda	136	-	39.7m	IQR [25.2-61.8m]	y	not tertiary	full article	patients suspected nephroblastoma	exclusion criteria clearly reportes	R	n	>4
98	Karpaga et al	2021	India	16	-	-	<18y	y	tertiary	abstract	patients diagnosed with neuroblastoma in oposmyoclonus syndrome	NS	R	NS	1
99	Eleno Beierbach et al.	2020	Argentina	61	-	1.3y	NS. paediatric	NS	NS	abstract	patients diagnosed with renal tumours at hospital between 2009-2019	NS	R	NS	1
100	Fang et al	2022	China	139	3.5y	-	7d-15y	y	tertiary	full article	patients treated for non-wilms renal tumours between 2008-2019	exclusion criteria clearly reported	R	y	>4
101	Ekuk et al	2023	Uganda	41	-	-	NS paediatrics	y	NS. multicentre	full article	patients diagnosed with wilms tumour between 2017-2021	exclusion criteria clearly reported	R	y	4
102	crocoli et al	2018	Italy	43	13.2y	-	7-18y	y	multicentre	full article	patients enrolled on national registry between 2000-2018	NS	R	y	3
103	Zekri et al.	2020	Egypt	18	-	48.5m	NS paediatrics	y	tertiary	full article	patients treated for adrenocortical carcinoma between 2007-2016	NS	R	NS	>4
104	Abosoudah et al.	2018	Saudi Arabia	266	-	37.6m	0.3-184.7m	y	multicentre	abstract	patients referred for treatment of wilms tumour between 2001-2011	exclusion criteria reports	R	NS	4
105	Assia-Zamora et al.	2019	United Kingdom	11	11y	-	3-15y	y	tertiary	abstract	patients referred with pancreatic tumours between 2001-2016	NS	R	NS	2
106	Cairo et al.	2022	United states	14	4.1y	-	1.78-9.42y	no	NS	full article	patients diagnosed with IVC thrombus associated abdominal malignancies between 2006-2017	exclusion criteria clear	R	NS	4
107	Ghafoor et al.	2020	Pakistan	84	38.9m	-	5.5m-11y	y	NS	full article	patients diagnosed and treated for wilms tumours between 2012-2019	exclusion criteria clear	R	y	>4
108	Zhang et al.	2021	China	45	42.5m	-	NS paediatrics	NS	Tertiary	full article	patients treated for wilms tumour rupture between 2008-2017	NS	R	NS	3
109	Zhi et al.	2021	China	132	-	-	0-12m	y	NS	full article	patients diagnosed with hepatoblastoma <12m between 2005-2019	NS	R	NS	1
110	Kurucu et al.	2020	Turkey	16	-	10y	2.5-13y	y	NS	full article	patients diagnosed with gastric lymphoma between 1972- 2019	inclusion criteria clear	R	NS	>4
111	Ravindranath et al.	2018	India	18	-	168m	36-228m	y	NS	abstract	patients diagnosed with GI lymphoma over 17years	NS	R	NS	>4

ID	Authors	Year	Country	n	mean age	median age	age range	presenting symptom/ prior to diagnosis	Institution status	Type of report	Study population	Sampling strategy	Study design*	Case definition and verification	Level of detail reported (no. of symptoms)
112	Singh et al	2022	India	28	-	31m	4-96m	y	NS, multicentre	full article	patients received radiotherapy for wilms tumour between 2015-2019	NS	P	NS	4
113	Wang et al.	2019	China	28	3.7m	-	0-10m	NS	NS	full article	patients stage 4s neuroblastoma between 2000-2018	NS	R	NS	>4
114	Mandal et al.	2022	India	32	-	-	0-44m	y	tertiary	full article	patients diagnosed with renal tumours between 2014-2021	inclusion criteria clearl reported	NS	y	>4
115	Mansfield et al.	2019	United States	240	-	7y	<1-30y	y	NS	full article	patients treated for renal tumours between 1950-2017	inclusion criteria clearly reported	R	y	>4
116	Kawano et al.	2022	Singapore	15	-	5m	6d-12y	y	NS	full article	patients treated for retroperitoneal teratoma at single institution between 198-2017	NS	R	y	>4
117	Dong et al.	2021	China	41	-	24m	3-108m	y	tertiary	full article	patients diagnosed with clear cell carcoma at single institution between 2008-2019	inclusion criteria clearly reported	R	y	>4
118	Kebudi et al.	2020	Turkey	11	-	52m	10m-15y	y	NS	abstract	patients diagnosed with female genital rhabdomyosarcoma between 1990-2019	NS	R	NS	3
119	Farooq et al.	2018	Pakistan	44	-	-	NS. paediatric	NS	NS	full article	patients diagnosed with wilms tumour to single institute between 2014-2016	exclusion criteria clearly reported	R	y	>4
120	Pereyaslov et al.	2019	Ukraine	42	-	-	NS. paediatric	NS	NS	abstract	patients treated for adrenal tumour at single institute	NS	R	NS	2
121	Jabbar et al.	2018	Pakistan	68	-	-	<18y	y	NS	full article	patients referred for an ultrasound to investigated an abdominal mass between 2017-2018	Clearly reported - convenience sampling technique	NS	NS	1
122	Cox et al.	2018	South Africa	12	-	6.5y	3.6-10.3y	NS	tertiary	full article	patients treated for intra-cardiac extension of wilms tumour at presentation between 1984-2016	Inclusion criteria clearly reported	R	NS	>4
123	Arthur et al.	2021	UK	88	11.8y	-	1-17y	y	tertiary	full article	patients treated for paediatric ovarian cancer between 1990-2018		NS	y	4
124	Ankunda et al.	2020	Sub-saharan Africa	29	3.4y	-	NS paediatrics	NS	NS	abstract	patients diagnosed with wilms tumours at single institute between 2019-2020	NS	R	NS	2
125	Yang et al.	2019	China	152	-	5.75m	NS paediatrics	y	NS	full article	patients treated surgically for retroperitoneal teratoma between 2000-2017	inclusion criteria and strategy clearly reported	R	NS	>4
126	Sraidi et al.	2021	Morocco	64	-	3y	NS paediatrics	y	NS	abstract	patients diagnosed with wilms tumour between 2015-2019		R	NS	1
127	Khan et al.	2019	India	23	4y	-	0.58-10Y	y	NS	full article	patients diagnosed with wilms tumour between 2010-2016	NS	R	NS	3
128	Ahmad et al.	2021	Saudi Arabia	36	-	36m	1-117m	y	tertiary	full article	patients diagnosed with wilms tumour between 2001-2015	exclusion criteria clealry reported	R	NS	3
129	Brener et al.	2023	Brazil	132	-	3y	2m-12y	y	NS	full article	patients diagnosed with wilms tumour between 2000-2021	NS	R	NS	>4
130	Herrera-Toro et al.	2019	Colombia	84	3y	46.5m	<15y	y	quaternary , multicentre	full article	patients diagnosed with wilms tumour between 2005-2017	exclusion criteria clealry reported	R	All except 4 cases confirmed with histopathological diagnosis	>4
131	Seminara et al.	2019	Argentina	46	43m	-	IQR[17-39m]	y	NS	full article	patients diagnosed with wilms tumours at single institute between 2000-2015	NS	R	y	4
132	Tanyildiz et al.	2018	Turkey	32	-	4 y	NS paediatrics	y	NS	abstract	patients treated for wilms tumours between 2002-2018	NS	R	NS	3
133	Illade et al.	2018	Spain	40	-	2.5y	4m-15y	y	tertiary	full article	patients managed for nephroblastona between 2002-2016	clearly reported inclusion criteria	R	y	>4

*Study design: P: Prospective; R: Retrospective; NS: Not specified
